# A Scoping Review on the Influence of Contextual Factors on Training Load in Adolescent Soccer Players: What Do We Know?

**DOI:** 10.3390/sports12070172

**Published:** 2024-06-24

**Authors:** Rick Nijland, Tynke Toering, Cameron G. Watson, Johan de Jong, Koen A. P. M. Lemmink

**Affiliations:** 1 School of Sports Studies, Hanze University of Applied Sciences Groningen, 9747 AS Groningen, The Netherlands; t.t.toering@pl.hanze.nl (T.T.); jo.de.jong@pl.hanze.nl (J.d.J.); 2 Department of Human Movement Sciences, University of Groningen, University Medical Center Groningen, 9713 GZ Groningen, The Netherlandsk.a.p.m.lemmink@umcg.nl (K.A.P.M.L.)

**Keywords:** youth, young adult, psychological stress, psychosocial factors, RPE, systematic review

## Abstract

This scoping review aimed to systematically explore the breadth and extent of the literature regarding the relationship between contextual factors (CFs) and training load (TL) in adolescent soccer players. Further aims included comprehending potential underlying mechanisms and identifying knowledge gaps. CFs were defined as factors not part of the main training process, such as the coach–athlete relationship and educational responsibilities. PubMed, EBSCO APA PsycINFO, Web of Science, ProQuest Dissertations & Theses A&I, and SportRxiv were searched. Studies involving adolescent soccer players that investigated the CF–TL relationship and measured TL indicators were deemed eligible. Seventeen studies were included, reflecting the limited number of articles published regarding the CF–TL relationship. CFs were mostly related to match-play (N = 13) and phase of the season (N = 7). Moreover, these factors appeared to affect TL. CF related to players’ personal environment (N = 3) were underrepresented in the reviewed studies. Overall, the CF–TL relationship appears to be rarely scrutinized. A likely cause for this lack of research is the segregation of the physiological and psychological research domains, where the CF–TL relationship is often speculated upon but not measured. Therefore, a holistic approach is warranted which also investigates the effect of personal environment, such as stressful life stress events, on TL.

## 1. Introduction

Physical prowess is important for adolescent soccer players to reach the elite level. At the highest level, players may play up to 50 matches per season, covering up to 14 km per match [1]. During the match, a high number of accelerations and decelerations, high-intensity running and sprinting are performed, indicating the high physical demands [1].

Physical conditioning is crucial to meet those demands and through meticulous planning of training load (TL), practitioners aim to optimize the physical training process. TL can be divided into external (EL) and internal load (IL) [2]. EL is defined as “what the athlete does and can be observed”, such as the distance covered [3]. IL is the psychophysiological response of the body during the training session [3,4]. IL is often measured using objective or subjective measures, such as heart rate (HR) or rate of perceived exertion (RPE). Moreover, IL is regarded as the main stimulus for training effects [4]. Next to IL, individual factors (IFs) and contextual factors (CFs) also affect TL [3,4]. IFs are characteristics of the player (e.g., genetics or training background). CFs are factors that are not part of the main (physical) training process (e.g., environmental, social, and cultural) and potentially influence the training process [3]. Therefore, CFs are factors that occur outside of (physical) training sessions [3]. It has been suggested that CFs may have a stronger impact on players than IFs due to a perceived lack of control when stressors originate from the social or cultural context [5]. When coaches do not consider the moderating effect of CFs, unforeseen adverse effects might occur, such as under- or overtraining, or illnesses and injuries [3,6,7,8]. Therefore, gaining further insight into the effect of CFs on TL is imperative to help practitioners optimize training programs and recovery.

For practitioners working with adolescent soccer players, CFs are particularly relevant and challenging since adolescence is considered a stressful developmental period characterized as a non-linear and multidimensional process [9,10,11]. During this phase in life, many psychosocial stressors emerge for adolescents which could originate within or outside sports [7,10,11]. For example, the coach–athlete relationship or match performance are known to be psychosocial stressors for adolescents originating from the sports context [9,11]. Alternatively, education and peer influence are potential psychosocial stressors outside sports [7,8,12]. These factors could affect IL and hence indicate to practitioners the need to modify EL to get players in an optimal state [6].

Although the link between CFs and TL has been suggested multiple times, there does not appear to be a single clear mechanistic underpinning but rather a myriad of suggested pathways [6,13]. This is understandable due to the broad definition of CFs [3,14]. For example, environmental factors such as temperature and humidity are well-studied and affect TL through mainly physiological processes [15]. On the other hand, psychosocial factors, such as academic stress and media attention, potentially cause stress, which theoretically influences TL [6,8]. Still, the mechanisms behind the relationship between psychosocial CFs and TL are not yet fully understood. This is partly due to the historical biomedical focus when TL was scrutinized [14,16]. Nevertheless, some theoretical models within the sports science literature offer more insight into the relationship between CFs and TL.

Several frameworks integrate psychosocial and physical stress. In their seminal article, Kenttä & Hassmén [17] propose the interactive and additive effects of psychosocial and physical stress, making up total stress. This apparent indication that total stress is not just the sum of stress from different sources supports the notion of psychosocial stress influencing TL. Other authors propose a similar linkage. Mellalieu et al. [7] advocate for the term ‘psychological load’ instead of stressors or demands to define a player’s total psychological demands and differentiate it from physical load. Both add up and potentially interact to determine the load of players [7]. This supports the premise of a complex system with inter-relationships between important CFs and TL, yet the precise mechanisms in this linkage remain unspecified [7,13].

Although normally used for the prediction and prevention of injuries, the injury stress model from Andersen and Williams [18] offers interesting leads concerning the potential mechanisms. In this model, psychosocial factors, such as life events, influence the stress response. Due to the stress response, physiological adaptations cause increased muscle tension, which in turn can disturb motor coordination and impair flexibility. Findings by Otter et al. [19] support this conjecture. The authors observed a reduction in the running economy of runners after a severe negative life event. Increased psychosocial stress, elevated cortisol concentrations, and impaired recovery likely caused this reduction [19]. Moreover, three weeks after the event, a higher oxygen uptake of the runners was recorded at a fixed EL during the second stage of a standardized submaximal test. Thus, it seems plausible that psychosocial stress affects TL, but the underlying mechanism is yet to be explored.

Finally, it is important to recognize the different origins and nature of psychosocial stressors because they might cause different psychological responses and require different interventions [20]. Psychosocial stress can generally be categorized as organizational, performance (competition), or personal [20,21]. These are demands related to the player’s soccer academy, competition, or (life) events outside of sport, respectively [7]. This is in line with the notion that a holistic approach is warranted when investigating talented athletes’ personal environment by including both their sporting and non-sporting experiences [22]. Within the aforementioned categories, different activities, actors, or organizations could act as potential sources of stress or stress mitigators [11,22]. For adolescent soccer players, integral aspects of their personal environment involve factors such as schools, parents, peers, match-play, teammates, and coaches [22,23]. These factors were also observed as potential stressors or stress mitigators for adolescents [8,11,24]. Nowadays, with the prevalence of social media, social evaluation is easier than before. This might be especially true for adolescents, for whom social media can appear to be indispensable in life and also play a prominent role in their sports experience [25]. Additionally, match-play can be an important CF since Reeves et al. [11] reported making errors during match-play and the accompanying social evaluation as prominent stressors for adolescent soccer players. For those working with adolescent soccer players, it seems imperative to adequately gauge the potential effect of important CFs for adolescent soccer players so they can then aim to align the different CFs to minimize potential negative and maximize positive effects [3,7,8]. Hence, this study will focus on players’ personal environment including both factors within and outside of sport.

The influence of CFs on TL is seen as an important part of the physical training process [3,6]. Still, knowledge regarding this relationship seems to be unclear but could offer important insights for practitioners. Therefore, the primary aim is to explore the breadth and extent of the literature regarding the relationship between CFs and TL for adolescent soccer players. A scoping review will be conducted, since this is particularly useful as it addresses exploratory research questions [26]. Furthermore, the second aim is to gain insight into potential mechanisms underlying the relation between CFs and TL. The final aim is to identify knowledge gaps within the scope of this review and provide suggestions for future research.

## 2. Materials and Methods

### 2.1. Identifying Relevant Studies

This review was registered on 13 December 2022, with Open Sciences Framework (https://doi.org/10.17605/OSF.IO/M8CEJ (15 May 2024)). The steps as suggested by the Joanna Briggs Institute [27] and Sabiston et al. [26] were followed. These steps included the following: (I) create and consult with a stakeholder group; (II) identify the research question(s); (III) identify relevant studies; (IV) create and register a protocol; (V) select and screen studies; (VI) chart the data; (VII) collate, summarize, and report the results; and (VIII) re-consult stakeholders and identify implications. Furthermore, the ’Preferred Reporting Items for Systematic Reviews and Meta-Analyses extension for scoping reviews’ (PRISMA-ScR) was used for this review (see Appendix A) [28]. An assessment of the risk of bias or methodological limitations is generally not performed unless compelling reasons related to the aim of the scoping review are presented [28]. The PCC mnemonic Population, Context, and Concept was followed to define the eligibility criteria [27]. Thus, studies involving adolescents with a mean age of 11–19 years old (population), within Association Soccer (context) that investigated the relationship between CFs and TL (concept) were included. The selected age range for adolescence is according to the definition of Salmela-Aro [10]. Moreover, the age range is in line with many European soccer academies and youth competitions (e.g., UEFA Youth League) which include teams up to Under-19. There was no limitation concerning years of publication. However, only articles in English were included.

The search strategy for scoping reviews ought to be comprehensive and include an initial search, subsequent modification of the search strategy, and a manual search of reference lists of included articles [27]. Before selecting keywords, a group of experts was consulted, and their input was used to conduct the initial search in Pubmed. Next, the key terms of the retrieved articles were analyzed. Thereafter, Pubmed, EBSCO APA PsycINFO, and Web of Science were searched based on the initial search and relation with the aim of the scoping review. As advised, grey literature was also included in this scoping review [26]. However, in line with the research question and the available time and resources, only dissertations and theses were searched. Grey literature was examined using ProQuest Dissertations & Theses A&I and SportRxiv due to their relevance. Finally, an information specialist experienced in literature reviews was consulted during the entire process.

The PCC criteria were used to determine the inclusion and exclusion of studies (see Table 1).

Search terms consisted of the main themes of the scoping review. The keywords and Boolean operators for the search were as follows:

1. Contextual factors
  (Context* OR “Contextual factor*” OR Situation* OR Environment* OR Ecological OR “Social load” OR “Social Stress” OR “Social support” OR “Psychological Stress” OR “Psychological Load” OR “Psychosocial stress” OR “Psychosocial Load” OR “daily life” OR “Daily lives” OR “ life stress” OR “life load” OR “Match related” OR “Match-related” OR “Match location” OR “Match outcome” OR Opposition Or Opponent* OR School OR Universit* OR College OR Academic* OR Education* OR Peer OR Peers OR Friend* OR Family OR Parent OR Parents OR Sibling* OR Coach* OR Staff OR Manager* OR Trainer* OR teammate* OR “Social Media” OR “Screen time” OR “dual career” OR “dual-career” OR Work OR profession OR occupation*)
2. Training load
  (“Training load” OR “Internal Load*” OR “External Load*” OR workload OR Load OR RPE OR dRPE OR “differential RPE” OR Exertion OR “Heart rate” OR TRIMP OR iTRIMP OR Speed OR Velocit* OR “Speed Zone*” OR Distance* OR Acceleration* OR Deceleration* OR Sprint* OR “high speed running” OR “very-high speed running” OR “very high speed running”)
3. Adolescent
  (Adolescen* OR Young OR Youth OR Talent* OR Junior OR Collegiate)
4. Soccer
  (Soccer OR Football OR “soccer player*” OR “football player*”)

Numbers 1, 2, 3, and 4 were combined with the AND Boolean operator. Furthermore, title and abstract terms were utilized to reduce noise in the search results since the initial search demonstrated substantial noise. The search terms were adapted specifically for each database and its command language, including MeSH terms and APA Thesaurus of Psychological Index Terms, and are provided in Appendix B, Appendix C, Appendix D, Appendix E and Appendix F. The search was conducted on 20 November 2023.

All references including abstracts were exported into Covidence systematic review software (Veritas Health Innovation, Melbourne, Australia). Duplicates were removed and the remaining papers were independently assessed by two researchers (R.N. and C.G.W.) using the aforementioned criteria. In case of conflict regarding an article inclusion/exclusion, a third reviewer (T.T.) made the final decision.

### 2.2. Consultation

Five experts were consulted for their expert opinions concerning the aims and research questions [26,27]. The stakeholder group consisted of 1 sports scientist embedded with a professional soccer club, 1 performance coach, 1 head of a youth soccer academy, 1 study counsellor, and 1 researcher appointed at a university. All experts had multiple years (5 to 20 years) of experience in or with adolescent soccer and were under contract by or worked together with a professional soccer academy (different clubs are involved). The stakeholders have been consulted during several stages [26,29]. Three engagements were held and were in line with the expert group’s situational preferences (e.g., location): (I) topic consultation and input meeting, (II) consistent involvement, and (III) reaction and dissemination meeting [29]. The first meeting was held before the study screening, the second meeting during the selecting studies and charting data phase, and the last meeting after drawing up the results.

### 2.3. Data Extraction

After study selection and screening for eligibility criteria, data were extracted from the full-text articles. The following information was extracted from the studies: author(s), year of publication, source origin, aims/purpose of the study, study population and sample size, methodology, type of contextual variables, TL variables, duration of the intervention, potential underlying mechanism(s), and key findings. The data extraction format was calibrated beforehand between two authors (R.N. and C.G.W.) and discussed with the rest of the authors.

## 3. Results

### 3.1. Literature Search

**Figure 1:**
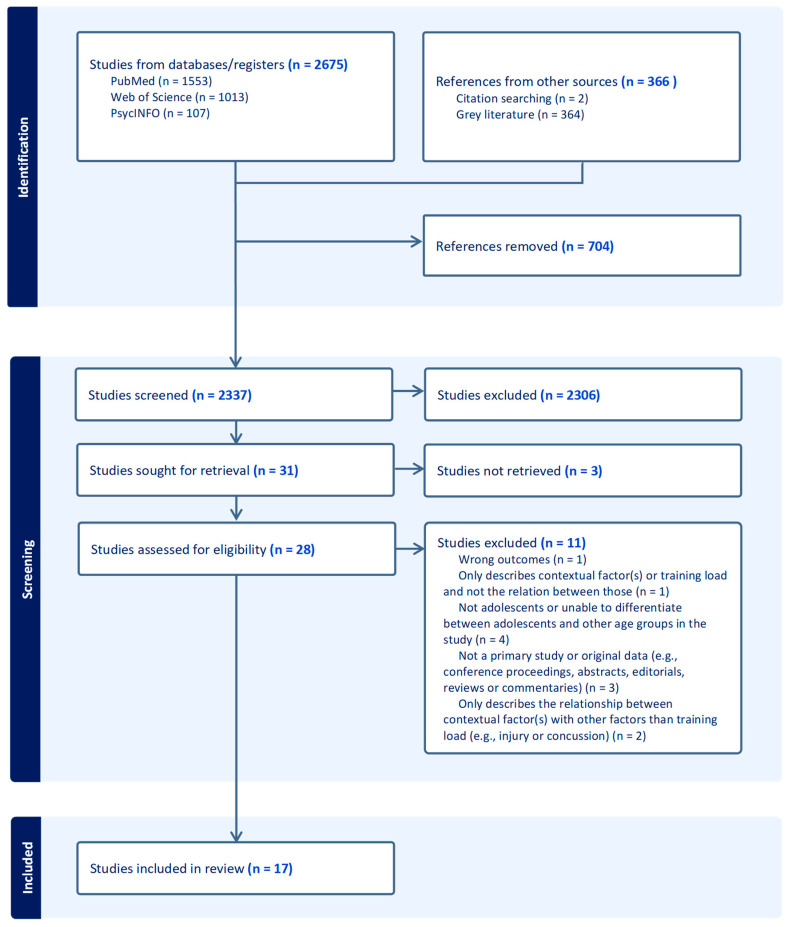
Flow chart for the article selection process of the Preferred Reporting Items for Systematic Reviews and Meta-analyses extension for Scoping Reviews (PRISMA-ScR).

The initial search identified 3041 articles of which 704 were duplicates. A total of 2337 studies were screened for title and abstract which resulted in thirty-one articles eligible for full-text screening. Fourteen articles were excluded according to predefined exclusion criteria (see Figure 1), leaving seventeen articles.

### 3.2. Sample Characteristics

Table 2 shows the sample characteristics of the included articles. The articles were published between 2008 and 2022 with most studies (N = 4) appearing in 2021. The origins of the studies were diverse, but most studies originated from the United States of America (N = 5). Only two studies (11.1%) recruited a female population. Moreover, in the included studies, the population’s mean age ranged from 13 years old to a maximum of 20 years old for collegiate players. Playing level was not categorized according to a standard most of the time and thus a diverse terminology of playing level was present, such as collegiate playing level (e.g., NCAA Division I) or elite. The included number of participants in the studies ranged from 13 to 107 with an average of 42.2 ± 31.1 participants. The duration of the studies varied from as short as one training session up to one season or calendar year.

### 3.3. Breadth and Extent of Relationship between Contextual Factors and Training Load

**Figure 2:**
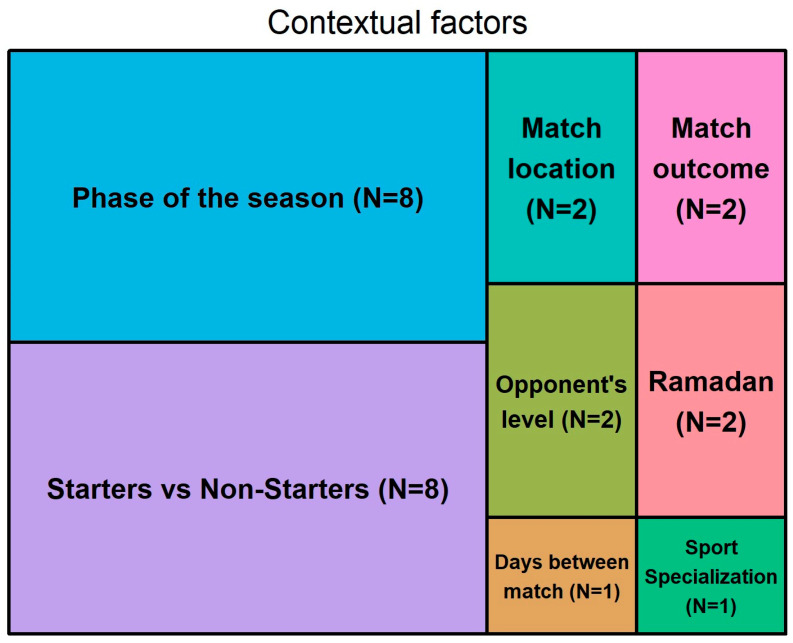
Treemap depicting the contextual factors and the total times they were investigated in studies.

Figure 2 depicts a tree map showing the number of times CFs have been included in a study. The phase of the season (N = 7) and starters vs non-starters (N = 7) had the highest number of times they were included in studies.

### 3.4. Co-Occurrence of Contextual Factors with Training Load Indicators

**Figure 3:**
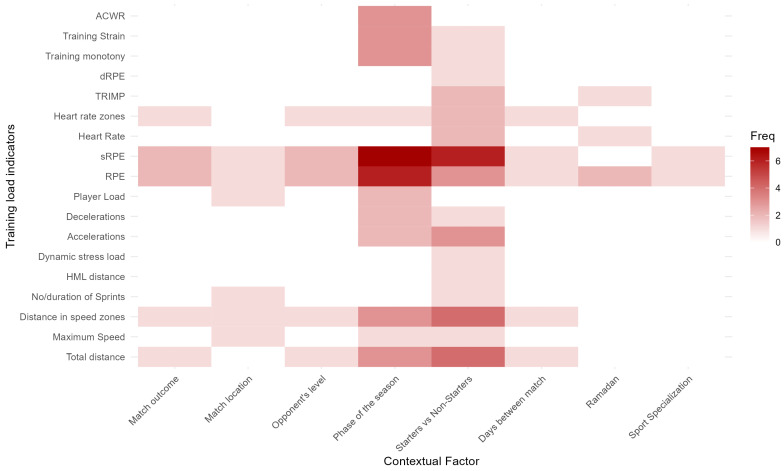
Heatmap of contextual factors and training load indicators.

Figure 3 depicts a heatmap of CFs and TL indicators. The heatmap indicates that RPE and session-RPE (sRPE) are the most used IL indicators. The most used EL indicators are the distance covered in different speed zones and the total distance covered.

### 3.5. Effect of Contextual Factors on Training Load

In general, CFs related to the match demonstrated an effect on TL in the following or preceding weeks. Most studies concerning starting status observed a difference between starters and non-starters in TL with starters accumulating a greater weekly TL [32,33,34,37,38,40]. However, when matches were excluded, the TL for non-starters was usually higher [32,38]. Other factors related to match-play, such as match location and opponent’s level show inconclusive effects on TL. Brito et al. [30] observed an increased TL after an away match, whereas Oliva-Lozano et al. [44] did not find any difference in TL due to match location. Similar results were found concerning the opponent’s level. Brito et al. [30] noted lower TL scores before and after playing against a top-level opponent. Contrasting these results, Curtis et al. [31] did not observe differences in TL due to the opponent’s relative strength. The aforementioned studies were more in line with each other when investigating match outcomes. Both studies found that TL during training increased after a loss [30,31].

TL appears to be influenced by the phase of the season. Studies that did include a pre-season phase found that during pre-season, the TL was highest compared to other phases of the season [31,45]. For the other phases of the season, the results were equivocal. For in-season phases, several studies observed that the mid-season had the highest or the equally highest TL [42,43,47]. Alternatively, Brito et al. [30] found that TL decreased throughout the season. In a mixed-design study, Pass et al. [45] found similar results but also noticed unintended distributions and fluctuations of TL across phases. It is important to recognize, however, that the included studies differ in methodology concerning the categorization of the seasonal phases. For instance, some studies have been conducted with American collegiate soccer where a season typically lasts from August until November, whereas in other regions a season could last almost one year.

Finally, other included CFs, Ramadan and sports specialization, did not seem to significantly influence TL in adolescent soccer players. Both studies concerning Ramadan were conducted by the same research group as part of a larger study and they found a nonsignificant marginally higher IL for fasting players [35,36]. Watson et al. [46] investigated the effect of sport specialization (i.e., participating in other sports than soccer) on TL and also did not find any significant effects on TL, but noticed a decreased sleep quality in specialized players.

### 3.6. Potential Underlying Mechanisms

All mechanistic underpinnings between the CFs and TL proposed by the authors of each study were related to the study’s topic. For example, for match-related factors (e.g., match location) periodization strategies and coaching experience were mentioned as potential mechanisms [31,32,33]. Specifically, for starting status, coaches try to compensate for the “missed” TL for non-starters [37,38,40,44]. Martins et al. [38] suggested that non-starters might try too hard to prove themselves and thus demonstrate higher TL. For Ramadan, the explanation was related to physiological changes due to hydration status or reduced sleep quantity and quality [35,36]. Interestingly, Pass et al. [45] noted that micropolitics within the organization and organizational demands could cause deviations within and from periodization. Stress was not specifically mentioned as a potential underlying mechanism in the reviewed studies, but one study mentioned the possibility of stress affecting the TL indices in U-14 players [42].

## 4. Discussion

### 4.1. General Discussion

CFs have long been theorized to affect TL [6,17]. It has been suggested to focus on CFs which potentially cause psychosocial stress as stress could be responsible for underpinning the relationship between CFs and TL [6,17,18]. Therefore, the primary aim was to explore the breadth and extent of the literature regarding the relationship between CFs and TL. Furthermore, we aimed to gain a deeper understanding of potential mechanisms and identify knowledge gaps. Despite the acknowledged potential importance of CFs related to TL, the main result of the scoping review was the dearth of articles (N = 17) found regarding this relationship. Furthermore, most articles focus on match-related factors or phases of the season. Starting status and phase of the season are likely to affect TL in adolescent soccer players. However, due to varying methodologies and results, the external validity beyond the included studies remains unclear. Most of the indicated underlying mechanisms were related to the CF investigated, such as coaching strategies and periodization to match-related CFs and micro-politics within the sporting environment to the phase of the season. Stress was not specifically mentioned as a pathway in the reviewed studies. Since there is a paucity of research concerning the relationship between CFs and TL, a significant gap in the literature has been identified.

The lack of research regarding the breadth and extent of the relationship between CFs and TL was somewhat surprising since it has been theorized that CFs affect TL [3,6,7,13]. Mainly factors related to the sports context, such as match-related factors (e.g., match outcome) and phase of the season have been investigated. Non-sport factors were restricted to Ramadan, indicating a major lack of research concerning adolescent players’ personal lives. One likely cause is the segregation between research domains. This probably led to a mainly unidimensional approach dividing psychological stress variables from research on physiology-related TL variables. In other words, studies related to this scoping review mostly focused on either CFs regarded as directly relevant for physiology-related TL variables while studies in the psychology domain do mention the possibility of a CF–TL relationship but do not measure it. To explore this view, we conducted an additional search in PubMed with the search terms for adolescents and soccer combined with CFs or TL. Although not all relevant, this yielded approximately 3300 and 2500 additional articles for CFs and TL, respectively, supporting our notion. Moreover, the number of studies found in PsycINFO, the leading international bibliography for psychology, only resulted in 107 hits. This demonstrates a wealth of research opportunities when adopting a holistic view to scrutinize TL. Additionally, the potential importance of CFs was corroborated by two members of the stakeholder’s group who indicated during the third session that from their experience, players’ TL is affected by personal factors such as their family life, peers, or dual-career challenges. Nevertheless, our experts found it difficult to adequately gauge the effect of the CFs and how to subsequently deal with them. Therefore, analogous to other authors who advocated for a holistic approach instead of reductionism to understand the dose–response relationship of TL, our results also indicate the need for more knowledge on the impact of CFs on TL [13,48,49]. By incorporating psychological indices to determine the psychological demands inside and outside of sport, a more comprehensive view can be obtained [7].

Opposite to theoretical and expert assumptions, stress was not mentioned as a potential mechanism in the reviewed studies [7,17]. Nevertheless, research shows that psychosocial stress could originate from players’ sporting and non-sporting context (e.g., education, match performance, family life) and has the potential to moderate TL [6,7,11]. This is in line with a recent study where coaches expressed the importance of four non-physical factors (coach–athlete relationship, life stress, athletes’ belief in the plan, and psychological and emotional stress) as integral for an athlete to physically adapt to a training plan [50]. Moreover, a severe negative life event impaired the running economy for sustained periods in runners [19]. Therefore, investigating the effect of life stress on TL could lead to new perspectives, more so because it has been noted that personal factors are an under-researched area with currently unclear effects [7]. Furthermore, accounting for other factors such as coping resources, personality, and history of stressors could be considered as they possibly modify the stress response [18]. Additionally, identifying the type, intensity, duration, and frequency of the stressors and linking those to daily TL could further our understanding of the (temporal) role of the environment and aid coaches in adopting a holistic approach when developing youths into elite players [7,48].

As expected, RPE and sRPE were the most used TL indicators overall. This is likely due to RPE being a valid, low-cost, and easy-to-administer measure [4]. Moreover, subjective measures are capable of reflecting the blended input of multiple sensory information channels and thus also capture the player–environment interaction [51]. However, without EL indicators, it would be more difficult to determine the effect CFs have on IL since IL is the psychophysiological response to EL [3,4]. A total of 10 out of 17 studies only included IL indicators. Therefore, future research should include both EL and IL indicators to gain a more comprehensive view regarding the influence of CFs on TL.

### 4.2. Limitations

This scoping review is not without limitations. First, it could be argued that the included CFs were not exhaustive. However, a consensus regarding the definition of CFs is lacking [14]. Moreover, the available literature indicated the included CFs as important for adolescent soccer players and they were linked with stress in general [11,22]. Furthermore, we asked experts for input regarding potentially relevant CFs which lined up with the selected CFs and therefore are confident the included CFs are relevant for practitioners.

Second, a definitive age range for adolescence is not universally specified because adolescence is mostly a social and not a biological construct [10]. In this review, the maximum mean age of adolescents was 19 years. However, some studies included in this review featured adolescents with a mean age of 20 years old. Although the age of adolescents was at the high end of the spectrum, we decided to still include those studies because they involved collegiate players, meaning players needed to combine education with soccer responsibilities like younger adolescents, providing useful information. Moreover, within certain national associations (e.g., the Dutch Royal Football Association) the women’s youth competition runs up to 20 years of age. Finally, adolescence is considered a phase of transition from child to adult and emerging adulthood has also been proposed as the latest phase of adolescence which runs from 18 until 25 years old [10]. Thus, by including these studies, additional relevant information regarding the final phases of adolescence is provided, which could be utilized by sports scientists and practitioners alike.

Third, by including only English-written articles the language of publication bias might be present. It is possible that relevant articles were missed. Nevertheless, a restriction based on the English language is common [26]. Moreover, the initial search did not yield relevant articles in other languages. Therefore, we expect the language of publication bias to be of limited influence on our results.

Finally, the generalizability of the results is likely limited to male adolescent soccer players. Our findings confirm that the female population is underrepresented in the literature. Therefore, generalizing these results to female adolescents should be done with caution.

## 5. Conclusions

The relationship between CFs and TL is a domain where a lot of research is yet to be done. Despite the theorized link and call for action of others, the assumption is that most research is restricted to their respective research disciplines investigating CFs or TL separately. Nonetheless, the available research shows that CFs can influence TL in adolescent soccer players. Specifically, performance-related CFs could cause unwanted alterations in TL. In the included studies, mechanistic underpinnings are mainly attributed to coaching strategies and periodization. Still, the role of stress has been hypothesized and could offer interesting avenues for future research, such as the effect of life stress on TL. Moreover, if we want to further understand the TL of adolescent soccer players, a holistic approach concerning TL and recovery management is vital. Adolescent soccer players do not live in a physical training vacuum but are part of their environment, which affects their physical training process.

## Figures and Tables

**Table 1:** Study inclusion and exclusion criteria.

Inclusion Criteria	Exclusion Criteria
Full text available	No full text available
English language	Not English language
Adolescents (mean age 11–19 years old) of either sex	Not adolescents or unable to differentiate between adolescents and other age groups in the study
Healthy subjects	Does not involve healthy subjects (e.g., injured or return-to-play)
Association Soccer	Other football codes than Association Soccer (e.g., Rugby)
Training load is (partly) measured during (part of) training sessions	Training load is not measured during (part of) training sessions (e.g., during matches)
Describing the relationship between a contextual factor and training load (external load and/or internal load)	Only describes contextual factor(s) or training load and not the relation between those
	Only describes the relationship between contextual factor(s) with other factors than training load (e.g., injury or concussion)
	Describes the relationship between contextual factors during a training session and training load
Primary study design with original data	Not a primary study or original data (e.g., conference proceedings, abstracts, editorials, reviews or commentaries)

**Table 2:** Sample characteristics and key findings of the included studies.

Reference	Country	Aim of the Study	General Methodology	Duration	Population Description	Key Findings
Brito et al., 2016 [30]	France	To examine match-related contextual factors concerning perceived TL and fatigue in soccer. To investigate the seasonal variation of self-reported TL and fatigue.	Observational design. Perceived exertion and fatigue were collected weekly.	1 season (2009–2010)	13 highly trained U-19 players (age 18.6 ± 0.5 yrs) from a first league club in France.	Higher TL scores after a defeat or a draw. Lower TL scores before and after matches against top-level opponents. Weekly TL was higher after an away match. General decrease in TL throughout the season.
Curtis et al., 2020 [31]	United States of America	To examine seven separate contextual factors’ influence on EL and IL in men’s collegiate soccer.	Observational design with four teams in total. EL and IL measures were collected.	August-November 2016 and 2017	107 NCAA Division I male collegiate soccer players (age 20 ± 2 yrs)	Season phase, days relative to match, days between match, and previous match outcome affected EL and IL. Opponents’ ranking and player’s role did not significantly affect average TL.
Curtis et al., 2021 [32]	United States of America	To quantify and compare seasonal total, match-, and training-accumulated TL by player status in men’s collegiate soccer.	Observational design. All training sessions and matches were captured for physical and physiological workloads.	August-November 2016 and 2017	107 NCAA Division I male collegiate soccer players (age 20 ± 2 yrs)	Starters accumulated more total physical and physiological workloads over the season.
Dalen et al., 2019 [33]	Norway	To investigate differences in physical and physiological in-season TL between starters and non-starters	Observational design. Players were monitored for TL during on-pitch training sessions and matches.	10 weeks (during the 2018 season)	18 junior soccer players (age 15.7 ± 0.5 yrs)	Starters had higher in-season physical external TL. Non-starters had a higher IL
Furtado Mesa et al., 2023 [34]	United States of America	To estimate workloads accumulated across one season between starters and non-starters	Observational design. All training sessions and official matches were monitored for TL.	August 2019–November 2019	19 NCAA Division I college female collegiate soccer players (age 20 ± 1.61 yrs)	Starters accumulated higher EL throughout the season. TL differed in matches, but not in training sessions.
Leiper et al., 2008 [35]	Tunisia	To investigate the psychosomatic factors in young Muslim football players during normal training and competitive schedules during Ramadan.	Observational design. A fasting and non-fasting group were monitored and tested over several weeks regarding multiple aspects (e.g., daily TL, physical testing, sleep quality).	7 weeks (during 2006 Ramadan)	87 Tunisian junior players from four teams from League 1 (N = 3) or League 3 (N = 1) clubs of the Tunisian National Senior Leagues (age 18 ± 1 yrs).	No consistent increase in measures for all players who participated in Ramadan.
Leiper et al., 2008 [36]	Tunisia	To determine whether the TL of fasting players was similar to that of non-fasting players.	Observational design. A fasting and non-fasting players were monitored for one training session during the third week of Ramadan. HR and RPE were measured.	One training session (during 2006 Ramadan)	79 young male football players from four Tunisian teams (age 18 ± 1 yrs)	TL of both groups were effectively similar during training, although the fasting group experienced marginally higher heart rates.
Los Arcos et al., 2017 [37]	Spain	To compare the respiratory and muscular perceived TL accumulation in elite-oriented young soccer players depending on starting status.	Observational design. Players were monitored regarding perceived TL. The competitive season was divided into 5 blocks of 6–8 weeks.	35 weeks (2012–2013)	24 young outfield professional soccer players (age 20.3 ± 2.0 yrs) from a reserve team of a Spanish La Liga club.	Respiratory and muscular perceived TL variations across the competition periods were limited for both groups. Starters accumulated greater perceived TL than non-starters due to match load. A progressive TL increase until mid-week and subsequent decrease until MD-1.
Martins et al., 2021 [38]	Portugal	To describe and compare the within-season variations of EL and IL indicators between starters and non-starters.	Observational design. IL was monitored. Only normal training sessions were included.	12 months from June 2017 to July 2018	17 U-17 soccer players from a Portuguese elite team: starters (age 16.2 ± 0.4 yrs) and non-starters (age 16.2 ± 0.2 yrs)	Significant differences between RPE and sRPE at MD and MD + 2 between groups. Some mesocycles provided a higher workload for non-starters. In general, IL indices were similar for starters vs. non-starters.
Maughan et al., 2022 [39]	Scotland	To quantify and describe the relationship between IL and EL variables across phases of the season.	Observational design. Both subjective and objective TL measures were obtained. Normal training sessions and matches were included.	47 weeks (2018/2019 season)	20 male youth soccer players (age 17.4 ± 1.3 yrs) from a professional Scottish club	Depending on seasonal phase, PCA revealed one or two components with several TL factors. Univariate measures are unlikely sufficient, and this limitation is affected by the phase of the season.
McLean et al., 2012 [40]	United States of America	To examine how Pmax changes during a female collegiate soccer season. Furthermore, to describe changes in perceptual fatigue responses during the in-season period.	Observational design. A women’s collegiate soccer team was monitored. Training sessions and matches were monitored using sRPE. Pmax was assessed nine times in-season.	16 weeks (1 season during the 2010 season)	19 division I female collegiate soccer players (age 19.9 ± 1.2 yrs).	Higher mean weekly load for starters compared to non-starters. Load was similar for starters and non-starters during training, except for week 1. Starters experienced significant reductions in Pmax during the second half of the season.
Nobari et al., 2020 [41]	Iran	To describe the weekly variations of TL metrics and well-being status and to analyse the associations between TL metrics and weekly reports of well-being status.	Observational design. Daily TL data were analysed to report changes in weekly load during the match season and wellness status.	20 weeks	29 young elite soccer players (age 15 ± 0.2 yrs from one team competing in the Iran national under-16 competitions.	Highest values of acute, chronic load and training strain in mid-season and the lowest values in early season. Highest values of accumulated weekly fatigue, stress, and DOMS in the end-season and the lowest values of sleep and stress in the early season.
Nobari et al., 2021 [42]	Iran	To describe daily TL throughout the competition season, analyse weekly differences among phases of the season and playing positions, and compare the TL variables over the competition period for the whole team.	Observational design. Daily monitoring was conducted. The competition was split into three macrocycles: early season, mid-season, and end-season.	26 weeks (1 season)	26 young male soccer players (age 13.3 ± 0.2 yrs) from one team competing in the Iran U-14 national team competitions.	Higher weekly monotony and training strain in mid-season. Weekly acute and chronic workload decreased from early to mid- and end-season.
Nobari et al., 2021 [43]	Iran	To investigate the variations of training workload in micro- and mesocycles, based on position.	Observational design. Daily player monitoring with the season divided into three mesocycles: early, mid-, and end-season.	20 weeks (1 season)	26 young soccer players (age 15.5 ± 0.2 yrs) playing in Iran’s U-16 competitions.	Only weekly chronic workload and training strain demonstrated differences in terms of the mesocycles. Highest values for multiple indices were detected in the mid-season and lowest during the early season.
Oliva-Lozano et al., 2020 [44]	Spain	To describe training and match activity demands profile in U-19 soccer players; to compare the profile depending on the type of session and differentiate between the profiles depending on the match location.	Observational design. Type of session and match location were used for comparison. Using Wireless Inertial Measurements Units, data were collected.	5 weeks (during 2017–2018 season)	25 U-19 soccer players (age 18.2 ± 0.87 yrs) from a U-19 team playing in the Spanish National League Championship.	No differences were found for match location in any variable regarding the activity-demands profile. Training sessions showed differences between weeks in TL. Moreover, players facing competition experienced higher TL in matches compared to training sessions.
Pass et al., 2022 [45]	England	To assess the TL experienced during pre-season and in-season mesocycles in youth soccer, and investigate how the intended periodized approach was implemented as planned.	Mixed-design study with two phases. Phase 1: Observational design. Four sessions were monitored for EL per seasonal phase. Phase 2: Multiple semi-structured interviews with the strength and conditioning coach.	One season (2018–2019)	Phase 1: 17 youth soccer players (age 17.2 ± 1.0 yrs) from a professional English Category 2 EPPP soccer academy. Phase 2: One Strength and conditioning coach	EL concerning training volume reduced during the seasons, with in-seasons 1 and 2 showing the largest values. EL concerning training intensity were highest during pre-season, which was deemed too high.
Watson et al., 2019 [46]	United States of America	To evaluate the relationships between sport specialization, sleep, and subjective well-being in female youth soccer players, while adjusting for the influence of TL and age.	Observational design. sRPE was collected together with sleep and well-being data.	4 month season	54 Female players from U-14 to U-18 teams within a community soccer organization (age 15.2 ± 1.5 yrs)	TL did not differ between specialized and non-specialized female players. Specialized players showed decreased sleep quality.

Abbreviations: DOMS, Delayed Onset of Muscle Soreness; EL, External Load; HR, Heart Rate; IL, Internal Load; MD, Match Day; NCAA, National Collegiate Athlete Association; PCA, Principal Component Analysis; Pmax, Maximal Power Output; RPE, Rating of Perceived Exertion; sRPE, session Rating of Perceived Exertion; TL, Training Load; yrs, years old.

## Data Availability

The original contributions presented in the study are included in the article, further inquiries can be directed to the corresponding author.

## Appendix A

**Table A1:** Preferred Reporting Items for Systematic reviews and Meta-Analyses extension for Scoping Reviews (PRISMA-ScR) Checklist.

SECTION	ITEM	PRISMA-ScR CHECKLIST ITEM	REPORTED ON PAGE #
**TITLE**			
Title	1	Identify the report as a scoping review.	# 1
**ABSTRACT**			
Structured summary	2	Provide a structured summary that includes (as applicable): background, objectives, eligibility criteria, sources of evidence, charting methods, results, and conclusions that relate to the review questions and objectives.	# 1
**INTRODUCTION**			
Rationale	3	Describe the rationale for the review in the context of what is already known. Explain why the review questions/objectives lend themselves to a scoping review approach.	# 1–3
Objectives	4	Provide an explicit statement of the questions and objectives being addressed with reference to their key elements (e.g., population or participants, concepts, and context) or other relevant key elements used to conceptualize the review questions and/or objectives.	# 3
**METHODS**			
Protocol and registration	5	Indicate whether a review protocol exists; state if and where it can be accessed (e.g., a web address), and if available, provide registration information, including the registration number.	# 3
Eligibility criteria	6	Specify characteristics of the sources of evidence used as eligibility criteria (e.g., years considered, language, and publication status) and provide a rationale.	# 4
Information sources *	7	Describe all information sources in the search (e.g., databases with dates of coverage and contact with authors to identify additional sources), as well as the date the most recent search was executed.	# 3–5
Search	8	Present the full electronic search strategy for at least one database, including any limits used, such that it could be repeated.	# 5/Appendix B, Appendix C, Appendix D, Appendix E and Appendix F
Selection of sources of evidence †	9	State the process for selecting sources of evidence (i.e., screening and eligibility) included in the scoping review.	# 4–5/Table 1
Data charting process ‡	10	Describe the methods of charting data from the included sources of evidence (e.g., calibrated forms or forms that have been tested by the team before their use, and whether data charting was done independently or in duplicate) and any processes for obtaining and confirming data from investigators.	#7
Data items	11	List and define all variables for which data were sought and any assumptions and simplifications made.	# 5
Critical appraisal of individual sources of evidence §	12	If carried out, provide a rationale for conducting a critical appraisal of included sources of evidence; describe the methods used and how this information was used in any data synthesis (if appropriate).	NA
Synthesis of results	13	Describe the methods of handling and summarizing the data that were charted.	# 7
**SECTION**	**ITEM**	**PRISMA-ScR CHECKLIST ITEM**	**REPORTED** **ON PAGE #**
**RESULTS**			
Selection of sources of evidence	14	Give numbers of sources of evidence screened, assessed for eligibility, and included in the review, with reasons for exclusions at each stage, ideally using a flow diagram.	# 6/Figure 1
Characteristics of sources of evidence	15	For each source of evidence, present characteristics for which data were charted and provide the citations.	# 8–12/Table 2
Critical appraisal within sources of evidence	16	If carried out, present data on critical appraisal of included sources of evidence (see item 12).	NA
Results of individual sources of evidence	17	For each included source of evidence, present the relevant data that were charted that relate to the review questions and objectives.	# 13–14
Synthesis of results	18	Summarize and/or present the charting results as they relate to the review questions and objectives.	# 14–15
**DISCUSSION**			
Summary of evidence	19	Summarize the main results (including an overview of concepts, themes, and types of evidence available), link to the review questions and objectives, and consider the relevance to key groups.	# 15
Limitations	20	Discuss the limitations of the scoping review process.	# 16
Conclusions	21	Provide a general interpretation of the results with respect to the review questions and objectives, as well as potential implications and/or next steps.	# 17
**FUNDING**			
Funding	22	Describe sources of funding for the included sources of evidence, as well as sources of funding for the scoping review. Describe the role of the funders of the scoping review.	# 17

JBI = Joanna Briggs Institute; PRISMA-ScR = Preferred Reporting Items for Systematic reviews and Meta-Analyses extension for Scoping Reviews. * Where *sources of evidence* (see second footnote) are compiled from, such as bibliographic databases, social media platforms, and websites. † A more inclusive/heterogeneous term used to account for the different types of evidence or data sources (e.g., quantitative and/or qualitative research, expert opinion, and policy documents) that may be eligible in a scoping review as opposed to only studies. This is not to be confused with *information sources* (see first footnote). ‡ The frameworks by Arksey and O’Malley (6) and Levac and colleagues (7) and the JBI guidance (4, 5) refer to the process of data extraction in a scoping review as data charting. § The process of systematically examining research evidence to assess its validity, results, and relevance before using it to inform a decision. This term is used for items 12 and 19 instead of “risk of bias” (which is more applicable to systematic reviews of interventions) to include and acknowledge the various sources of evidence that may be used in a scoping review (e.g., quantitative and/or qualitative research, expert opinion, and policy document). *From:* [28].

## Appendix B

In Table A2 the search query for EBSCO APA PsycINFO.

**Table A2:** Search strategy EBSCO APA PsycINFO.

#	Query	Limiters/Expanders
S104	S63 AND S87 AND S96 AND S102	Expanders-Apply equivalent subjectsNarrow by Language: englishSearch modes-Boolean/Phrase
S103	S63 AND S87 AND S96 AND S102	Expanders-Apply equivalent subjectsSearch modes-Boolean/Phrase
S102	S97 OR S98 OR S99 OR S100 OR S101	Expanders-Apply equivalent subjectsSearch modes-Boolean/Phrase
S101	DE “Soccer”	Expanders-Apply equivalent subjectsSearch modes-Boolean/Phrase
S100	(AB football players OR AB “football player*”) OR (TI football players OR TI “football player*”)	Expanders-Apply equivalent subjectsSearch modes-Boolean/Phrase
S99	(AB (soccer players or soccer athletes) OR AB “soccer player*”) OR (TI (soccer players or soccer athletes) OR TI “soccer player*”)	Expanders-Apply equivalent subjectsSearch modes-Boolean/Phrase
S98	AB football or TI football	Expanders-Apply equivalent subjectsSearch modes-Boolean/Phrase
S97	AB soccer OR TI soccer	Expanders-Apply equivalent subjectsSearch modes-Boolean/Phrase
S96	S88 OR S89 OR S90 OR S91 OR S92 OR S93 OR S94 OR S95	Expanders-Apply equivalent subjectsSearch modes-Boolean/Phrase
S95	DE “Gifted” OR DE “Savants”	Expanders-Apply equivalent subjectsSearch modes-Boolean/Phrase
S94	DE “Early Adolescence”	Expanders-Apply equivalent subjectsSearch modes-Boolean/Phrase
S93	AB collegiate OR TI collegiate	Expanders-Apply equivalent subjectsSearch modes-Boolean/Phrase
S92	AB junior OR TI junior	Expanders-Apply equivalent subjectsSearch modes-Boolean/Phrase
S91	AB talent* OR TI talent*	Expanders-Apply equivalent subjectsSearch modes-Boolean/Phrase
S90	AB youth OR TI youth	Expanders-Apply equivalent subjectsSearch modes-Boolean/Phrase
S89	AB young OR TI young	Expanders-Apply equivalent subjectsSearch modes-Boolean/Phrase
S88	AB adolescen* OR TI adolescen*	Expanders-Apply equivalent subjectsSearch modes-Boolean/Phrase
S87	S64 OR S65 OR S66 OR S67 OR S68 OR S69 OR S70 OR S71 OR S72 OR S73 OR S74 OR S75 OR S76 OR S77 OR S78 OR S79 OR S80 OR S81 OR S82 OR S83 OR S84 OR S85 OR S86	Expanders-Apply equivalent subjectsSearch modes-Boolean/Phrase
S86	DE “Velocity”	Expanders-Apply equivalent subjectsSearch modes-Boolean/Phrase
S85	DE “Heart Rate” OR DE “Heart Rate Variability” OR DE “Heart Rate Variability”	Expanders-Apply equivalent subjectsSearch modes-Boolean/Phrase
S84	DE “Perceived Stress”	Expanders-Apply equivalent subjectsSearch modes-Boolean/Phrase
S83	DE “Work Load”	Expanders-Apply equivalent subjectsSearch modes-Boolean/Phrase
S82	AB “very-high speed running” OR TI “very-high speed running”	Expanders-Apply equivalent subjectsSearch modes-Boolean/Phrase
S81	AB “high speed running” OR TI “high speed running”	Expanders-Apply equivalent subjectsSearch modes-Boolean/Phrase
S80	AB sprint* OR TI sprint*	Expanders-Apply equivalent subjectsSearch modes-Boolean/Phrase
S79	AB deceleration* OR TI deceleration*	Expanders-Apply equivalent subjectsSearch modes-Boolean/Phrase
S78	AB acceleration* OR TI acceleration*	Expanders-Apply equivalent subjectsSearch modes-Boolean/Phrase
S77	AB distance* OR TI distance*	Expanders-Apply equivalent subjectsSearch modes-Boolean/Phrase
S76	AB “speed zone*” OR TI “speed zone*”	Expanders-Apply equivalent subjectsSearch modes-Boolean/Phrase
S75	AB velocit* OR TI velocit*	Expanders-Apply equivalent subjectsSearch modes-Boolean/Phrase
S74	AB speed OR TI speed	Expanders-Apply equivalent subjectsSearch modes-Boolean/Phrase
S73	AB itrimp OR TI itrimp	Expanders-Apply equivalent subjectsSearch modes-Boolean/Phrase
S72	AB trimp OR TI trimp	Expanders-Apply equivalent subjectsSearch modes-Boolean/Phrase
S71	AB heart rate OR TI heart rate	Expanders-Apply equivalent subjectsSearch modes-Boolean/Phrase
S70	AB exertion OR TI exertion	Expanders-Apply equivalent subjectsSearch modes-Boolean/Phrase
S69	AB drpe OR TI drpe	Expanders-Apply equivalent subjectsSearch modes-Boolean/Phrase
S68	AB “differential rpe” OR TI “differential rpe”	Expanders-Apply equivalent subjectsSearch modes-Boolean/Phrase
S67	(AB (rpe or rate of perceived exertion or perceived exertion or borg scale)) OR (TI (rpe or rate of perceived exertion or perceived exertion or borg scale))	Expanders-Apply equivalent subjectsSearch modes-Boolean/Phrase
S66	AB load OR TI load	Expanders-Apply equivalent subjectsSearch modes-Boolean/Phrase
S65	(AB (internal load or external load or workload or physical demands or activity profile)) OR (TI (internal load or external load or workload or physical demands or activity profile))	Expanders-Apply equivalent subjectsSearch modes-Boolean/Phrase
S64	(AB (training load or training intensity or training volume) OR AB “training load”) OR (TI (training load or training intensity or training volume) OR AB “training load”)	Expanders-Apply equivalent subjectsSearch modes-Boolean/Phrase
S63	S1 OR S2 OR S3 OR S4 OR S5 OR S6 OR S7 OR S8 OR S9 OR S10 OR S11 OR S12 OR S13 OR S14 OR S15 OR S16 OR S17 OR S18 OR S19 OR S20 OR S21 OR S22 OR S23 OR S24 OR S25 OR S26 OR S27 OR S28 OR S29 OR S30 OR S31 OR S32 OR S33 OR S34 OR S35 OR S36 OR S37 OR S38 OR S39 OR S40 OR S41 OR S42 OR S43 OR S44 OR S45 OR S46 OR S47 OR S48 OR S49 OR S50 OR S51 OR S52 OR S53 OR S54 OR S55 OR S56 OR S57 OR S58 OR S59 OR S60 OR S61 OR S62	Expanders-Apply equivalent subjectsSearch modes-Boolean/Phrase
S62	DE “Occupations” OR DE “Employment Status” OR DE “Job Characteristics” OR DE “Job Search” OR DE “Nontraditional Careers” OR DE “Occupational Choice” OR DE “Occupational Mobility” OR DE “Occupational Tenure”	Expanders-Apply equivalent subjectsSearch modes-Boolean/Phrase
S61	DE “Dual Careers”	Expanders-Apply equivalent subjectsSearch modes-Boolean/Phrase
S60	DE “Screen Time”	Expanders-Apply equivalent subjectsSearch modes-Boolean/Phrase
S59	DE “Social Media” OR DE “Online Social Networks”	Expanders-Apply equivalent subjectsSearch modes-Boolean/Phrase
S58	DE “Medical Personnel” OR DE “Dentists” OR DE “Military Medical Personnel” OR DE “Nurses” OR DE “Optometrists” OR DE “Pharmacists” OR DE “Physical Therapists” OR DE “Physicians” OR DE “Psychiatric Hospital Staff”	Expanders-Apply equivalent subjectsSearch modes-Boolean/Phrase
S57	DE “Coaching” OR DE “Coaches” OR DE “Executive Coaching” OR DE “Life Coaching” OR DE “Sports Coaching” OR DE “Test Coaching” OR DE “Coaches”	Expanders-Apply equivalent subjectsSearch modes-Boolean/Phrase
S56	DE “Siblings” OR DE “Brothers” OR DE “Multiple Births” OR DE “Sisters” OR DE “Only Children” OR DE “Sibling Relations”	Expanders-Apply equivalent subjectsSearch modes-Boolean/Phrase
S55	DE “Parents” OR DE “Adoptive Parents” OR DE “Expectant Parents” OR DE “Fathers” OR DE “Foster Parents” OR DE “Homosexual Parents” OR DE “Mothers” OR DE “Parental Characteristics” OR DE “Single Parents” OR DE “Stepparents” OR DE “Surrogate Parents (Humans)”	Expanders-Apply equivalent subjectsSearch modes-Boolean/Phrase
S54	DE “Family” OR DE “Biological Family” OR DE” Dual Careers” OR DE “Dysfunctional Family” OR DE “Extended Family” OR DE “Family Background” OR DE “Family History” OR DE “Family Members” OR DE “Family of Origin” OR DE “Family Relations” OR DE “Family Resemblance” OR DE “Family Structure” OR DE “Family Work Relationship” OR DE “Interethnic Family” OR DE “Interracial Family” OR DE “Military Families” OR DE “Nepotism” OR DE “Nuclear Family” OR DE “Schizophrenia” OR DE “Stepfamily” OR DE “Family Relations” OR DE “Child Discipline” OR DE “Childrearing Practices” OR DE “Family Conflict” OR DE “Family Separation” OR DE “Intergenerational Relations” OR DE “Marital Relations” OR DE “Parent Child Relations” OR DE “Parental Role” OR DE “Sibling Relations” OR DE “Transgenerational Patterns” OR DE “Biological Family”	Expanders-Apply equivalent subjectsSearch modes-Boolean/Phrase
S53	DE “Friendship”	Expanders-Apply equivalent subjectsSearch modes-Boolean/Phrase
S52	DE “Peer Evaluation” OR DE “Peer Tutoring” OR DE “Peer Relations” OR DE “Peer Pressure” OR DE “Peer Pressure” OR DE “Peers” OR DE “Significant Others”	Expanders-Apply equivalent subjectsSearch modes-Boolean/Phrase
S51	(DE “Academic Stress” OR DE “Academic Environment” OR DE “Classroom Environment” OR DE “Same Sex Education” OR DE “School Environment”) OR (DE “Academic Settings” OR DE “Schools”)	Expanders-Apply equivalent subjectsSearch modes-Boolean/Phrase
S50	DE “Education” OR DE “Academic Settings” OR DE “Academic Specialization” OR DE “Adult Education” OR DE “Bilingual Education” OR DE “Client Education” OR DE “Coeducation” OR DE “Consumer Education” OR DE “Counselor Education” OR DE “Curriculum” OR DE “Death Education” OR DE “Distance Education” OR DE “Education Policy” OR DE “Educational Degrees” OR DE “Educational Financial Assistance” OR DE “Educational Placement” OR DE “Educational Programs” OR DE “Educational Quality” OR DE “Educational Reform” OR DE “Educational Standards” OR DE “Elementary Education” OR DE “Environmental Education” OR DE “Family Life Education” OR DE “Gifted Education” OR DE “Grade Level” OR DE “High School Education” OR DE “Higher Education” OR DE “Homework” OR DE “Middle School Education” OR DE “Multicultural Education” OR DE “Nontraditional Education” OR DE “Nursing Education” OR DE “Paraprofessional Education” OR DE “Personnel Training” OR DE “Preschool Education” OR DE “Private School Education” OR DE “Public School Education” OR DE “Religious Education” OR DE “Remedial Education” OR DE “School Attendance” OR DE “School Enrollment” OR DE “School Graduation” OR DE “School Readiness” OR DE “School Retention” OR DE “School Transition” OR DE “Secondary Education” OR DE “Social Work Education” OR DE “Special Education” OR DE “STEM” OR DE “Student Admission Criteria” OR DE “Student Records” OR DE “Teacher Education”	Expanders-Apply equivalent subjectsSearch modes-Boolean/Phrase
S49	DE “College Environment”	Expanders-Apply equivalent subjectsSearch modes-Boolean/Phrase
S48	DE “Psychosocial Factors” OR DE “Protective Factors” OR DE “Psychodynamics” OR DE “Psychosocial Outcomes” OR DE “Risk Factors”	Expanders-Apply equivalent subjectsSearch modes-Boolean/Phrase
S47	DE “Psychological Stress”	Expanders-Apply equivalent subjectsSearch modes-Boolean/Phrase
S46	DE “Social Support”	Expanders-Apply equivalent subjectsSearch modes-Boolean/Phrase
S45	DE “Social Stress”	Expanders-Apply equivalent subjectsSearch modes-Boolean/Phrase
S44	DE “Daily Activities” OR DE “Leisure Time”	Expanders-Apply equivalent subjectsSearch modes-Boolean/Phrase
S43	DE “Ecological Factors” OR DE “Pollution” OR DE “Topography”	Expanders-Apply equivalent subjectsSearch modes-Boolean/Phrase
S42	DE “Environment” OR DE “Built Environment” OR DE “Environmental Enrichment” OR DE “Environmental Planning” OR DE “Facility Environment” OR DE “Learning Environment” OR DE “Nature (Environment)” OR DE “Person Environment Fit” OR DE “Physical Comfort” OR DE “Public Space” OR DE “Single Sex Environments” OR DE “Social Environments” OR DE “Sustainability” OR DE “Therapeutic Environment” OR DE “Virtual Environment”	Expanders-Apply equivalent subjectsSearch modes-Boolean/Phrase
S41	AB profession	Expanders-Apply equivalent subjectsSearch modes-Boolean/Phrase
S40	AB work OR TI work	Expanders-Apply equivalent subjectsSearch modes-Boolean/Phrase
S39	AB “dual-career” OR TI “dual-career”	Expanders-Apply equivalent subjectsSearch modes-Boolean/Phrase
S38	AB “screen time” OR TI “screen time”	Expanders-Apply equivalent subjectsSearch modes-Boolean/Phrase
S37	AB “social media” OR TI “social media”	Expanders-Apply equivalent subjectsSearch modes-Boolean/Phrase
S36	AB teammate* OR TI teammate*	Expanders-Apply equivalent subjectsSearch modes-Boolean/Phrase
S35	AB trainer* OR TI trainer*	Expanders-Apply equivalent subjectsSearch modes-Boolean/Phrase
S34	AB manager* OR TI manager*	Expanders-Apply equivalent subjectsSearch modes-Boolean/Phrase
S33	AB staff OR TI staff	Expanders-Apply equivalent subjectsSearch modes-Boolean/Phrase
S32	AB coach* OR TI coach*	Expanders-Apply equivalent subjectsSearch modes-Boolean/Phrase
S31	AB sibling* OR TI sibling*	Expanders-Apply equivalent subjectsSearch modes-Boolean/Phrase
S30	AB parents OR TI parents	Expanders-Apply equivalent subjectsSearch modes-Boolean/Phrase
S29	AB parent OR TI parent	Expanders-Apply equivalent subjectsSearch modes-Boolean/Phrase
S28	AB family OR TI family	Expanders-Apply equivalent subjectsSearch modes-Boolean/Phrase
S27	AB friend* OR TI friend*	Expanders-Apply equivalent subjectsSearch modes-Boolean/Phrase
S26	AB peers OR TI peers	Expanders-Apply equivalent subjectsSearch modes-Boolean/Phrase
S25	AB peer OR TI peer	Expanders-Apply equivalent subjectsSearch modes-Boolean/Phrase
S24	(AB education* OR AB (education or school or learning or teaching or classroom or education system)) OR (TI education* OR TI (education or school or learning or teaching or classroom or education system))	Expanders-Apply equivalent subjectsSearch modes-Boolean/Phrase
S23	AB academic* OR TI academic*	Expanders-Apply equivalent subjectsSearch modes-Boolean/Phrase
S22	AB college OR TI college	Expanders-Apply equivalent subjectsSearch modes-Boolean/Phrase
S21	AB universit* OR TI universit*	Expanders-Apply equivalent subjectsSearch modes-Boolean/Phrase
S20	AB school OR TI school	Expanders-Apply equivalent subjectsSearch modes-Boolean/Phrase
S19	AB “opponent*” OR TI “opponent*”	Expanders-Apply equivalent subjectsSearch modes-Boolean/Phrase
S18	AB “opposition” OR TI “opposition”	Expanders-Apply equivalent subjectsSearch modes-Boolean/Phrase
S17	AB “match outcome” OR TI “match outcome”	Expanders-Apply equivalent subjectsSearch modes-Boolean/Phrase
S16	AB “match location” OR TI “match location”	Expanders-Apply equivalent subjectsSearch modes-Boolean/Phrase
S15	AB “match-related” OR TI “match-related”	Expanders-Apply equivalent subjectsSearch modes-Boolean/Phrase
S14	AB “life load” OR TI “life load”	Expanders-Apply equivalent subjectsSearch modes-Boolean/Phrase
S13	AB “psychosocial load” OR TI “psychosocial load”	Expanders-Apply equivalent subjectsSearch modes-Boolean/Phrase
S12	AB “psychosocial stress” OR TI “psychosocial stress”	Expanders-Apply equivalent subjectsSearch modes-Boolean/Phrase
S11	AB “psychological load” OR TI “psychological load”	Expanders-Apply equivalent subjectsSearch modes-Boolean/Phrase
S10	AB “psychological stress” OR TI “psychological stress”	Expanders-Apply equivalent subjectsSearch modes-Boolean/Phrase
S9	(AB (daily life or everyday life or daily activities or activities of daily living) OR AB daily life) OR (TI (daily life or everyday life or daily activities or activities of daily living) OR TI daily life)	Expanders-Apply equivalent subjectsSearch modes-Boolean/Phrase
S8	(AB (social support or social networks or social relationships or social inclusion or social exclusion or social isolation)) OR (TI (social support or social networks or social relationships or social inclusion or social exclusion or social isolation))	Expanders-Apply equivalent subjectsSearch modes-Boolean/Phrase
S7	AB “social stress” OR TI “social stress”	Expanders-Apply equivalent subjectsSearch modes-Boolean/Phrase
S6	AB “social load” OR TI “social load”	Expanders-Apply equivalent subjectsSearch modes-Boolean/Phrase
S5	AB ecological OR TI ecological	Expanders-Apply equivalent subjectsSearch modes-Boolean/Phrase
S4	AB environmental OR AB evironment* OR TI environmental OR TI evironment*	Expanders-Apply equivalent subjectsSearch modes-Boolean/Phrase
S3	AB situation* OR TI situation*	Expanders-Apply equivalent subjectsSearch modes-Boolean/Phrase
S2	AB contextual factors OR AB “contextual factor*” OR TI contextual factors OR TI “contextual factor*”	Expanders-Apply equivalent subjectsSearch modes-Boolean/Phrase
S1	AB context* OR TI context*	Expanders-Apply equivalent subjectsSearch modes-Boolean/Phrase

## Appendix C

Below is the search string for the database Web of Science.

TI=((Context* OR “Contextual factor*” OR Situation* OR Environment* OR Ecological OR “Social load” OR “Social Stress” OR “Social support” OR “Psychological Stress” OR “Psychological Load” OR “Psychosocial stress” OR “Psychosocial Load” OR “daily life” OR “Daily lives” OR “life stress” OR “life load” OR “Match related” OR “Match-related” OR “Match location” OR “Match outcome” OR Opposition Or Opponent* OR School OR Universit* OR College OR Academic* OR Education* OR Peer OR Peers OR Friend* OR Family OR Parent OR Parents OR Sibling* OR Coach* OR Staff OR Manager* OR Trainer* OR teammate* OR “Social Media” OR “Screen time” OR “dual career” OR “dual-career” OR Work OR profession OR “Social environment”[Mesh] OR Environment[Mesh] OR “Social support”[Mesh] OR Schools[Mesh] OR Universities[Mesh] OR Education[Mesh] OR “Peer group”[Mesh] OR “Peer Influence”[Mesh] OR Friends[Mesh] OR Family[Mesh] OR Parents[Mesh] OR Siblings[Mesh] OR “Social Media”[Mesh] OR “Screen Time”[Mesh] OR Work[Mesh] OR Occupations[Mesh] OR “Stress, Psychological”[Mesh] OR “Stress, Physiological”[Mesh]) AND (“Training load” OR “Internal Load*” OR “External Load*” OR Load OR RPE OR dRPE OR “differential RPE” OR Exertion OR “Heart rate” OR TRIMP OR iTRIMP OR Speed OR Velocit* OR “Speed Zone*” OR Distance* OR Acceleration* OR Deceleration* OR Sprint* OR “high speed running” OR “very-high speed running” OR “very high speed running” OR Workload[Mesh] OR “Physical Exertion”[Mesh] OR “Heart Rate”[Mesh] OR Acceleration[Mesh] OR Deceleration[Mesh] OR Running[Mesh]) AND (Adolescen* OR Young OR Youth OR Talent* OR Junior OR Collegiate OR adolescent[Mesh] OR “Young Adult”[Mesh]) AND (Soccer OR Football OR “soccer player*” OR “football player*” OR Soccer[Mesh])) OR AB=((Context* OR “Contextual factor*” OR Situation* OR Environment* OR Ecological OR “Social load” OR “Social Stress” OR “Social support” OR “Psychological Stress” OR “Psychological Load” OR “Psychosocial stress” OR “Psychosocial Load” OR “daily life” OR “Daily lives” OR “life stress” OR “life load” OR “Match related” OR “Match-related” OR “Match location” OR “Match outcome” OR Opposition Or Opponent* OR School OR Universit* OR College OR Academic* OR Education* OR Peer OR Peers OR Friend* OR Family OR Parent OR Parents OR Sibling* OR Coach* OR Staff OR Manager* OR Trainer* OR teammate* OR “Social Media” OR “Screen time” OR “dual career” OR “dual-career” OR Work OR profession OR “Social environment”[Mesh] OR Environment[Mesh] OR “Social support”[Mesh] OR Schools[Mesh] OR Universities[Mesh] OR Education[Mesh] OR “Peer group”[Mesh] OR “Peer Influence”[Mesh] OR Friends[Mesh] OR Family[Mesh] OR Parents[Mesh] OR Siblings[Mesh] OR “Social Media”[Mesh] OR “Screen Time”[Mesh] OR Work[Mesh] OR Occupations[Mesh] OR “Stress, Psychological”[Mesh] OR “Stress, Physiological”[Mesh]) AND (“Training load” OR “Internal Load*” OR “External Load*” OR Load OR RPE OR dRPE OR “differential RPE” OR Exertion OR “Heart rate” OR TRIMP OR iTRIMP OR Speed OR Velocit* OR “Speed Zone*” OR Distance* OR Acceleration* OR Deceleration* OR Sprint* OR “high speed running” OR “very-high speed running” OR “very high speed running” OR Workload[Mesh] OR “Physical Exertion”[Mesh] OR “Heart Rate”[Mesh] OR Acceleration[Mesh] OR Deceleration[Mesh] OR Running[Mesh]) AND (Adolescen* OR Young OR Youth OR Talent* OR Junior OR Collegiate OR adolescent[Mesh] OR “Young Adult”[Mesh]) AND (Soccer OR Football OR “soccer player*” OR “football player*” OR Soccer[Mesh]))

## Appendix D

Below is the search string for Pubmed.

(Context*[tiab] OR “Contextual factor*”[tiab] OR Situation*[tiab] OR Environment*[tiab] OR Ecological[tiab] OR “Social load” [tiab] OR “Social Stress” [tiab] OR “Social support”[tiab] OR “Psychological Stress”[tiab] OR “Psychological Load”[tiab] OR “Psychosocial stress”[tiab] OR “Psychosocial Load”[tiab] OR “daily life”[tiab] OR “Daily lives”[tiab] OR “life stress”[tiab] OR “life load”[tiab] OR “Match related”[tiab] OR “Match-related”[tiab] OR “Match location”[tiab] OR “Match outcome”[tiab] OR Opposition[tiab] Or Opponent*[tiab] OR School[tiab] OR Universit*[tiab] OR College[tiab] OR Academic*[tiab] OR Education*[tiab] OR Peer[tiab] OR Peers[tiab] OR Friend*[tiab] OR Family[tiab] OR Parent[tiab] OR Parents[tiab] OR Sibling*[tiab] OR Coach*[tiab] OR Staff[tiab] OR Manager*[tiab] OR Trainer*[tiab] OR teammate*[tiab] OR “Social Media”[tiab] OR “Screen time”[tiab] OR “dual career”[tiab] OR “dual-career”[tiab] OR Work[tiab] OR profession[tiab] OR “Social environment”[Mesh] OR Environment[Mesh] OR “Social support”[Mesh] OR Schools[Mesh] OR Universities[Mesh] OR Education[Mesh] OR “Peer group”[Mesh] OR “Peer Influence”[Mesh] OR Friends[Mesh] OR Family[Mesh] OR Parents[Mesh] OR Siblings[Mesh] OR “Social Media”[Mesh] OR “Screen Time”[Mesh] OR Work[Mesh] OR Occupations[Mesh] OR “Stress, Psychological”[Mesh] OR “Stress, Physiological”[Mesh]) AND (“Training load”[tiab] OR “Internal Load*”[tiab] OR “External Load*”[tiab] OR Load[tiab] OR RPE[tiab] OR dRPE[tiab] OR “differential RPE”[tiab] OR Exertion[tiab] OR “Heart rate”[tiab] OR TRIMP[tiab] OR iTRIMP[tiab] OR Speed[tiab] OR Velocit*[tiab] OR “Speed Zone*”[tiab] OR Distance*[tiab] OR Acceleration*[tiab] OR Deceleration*[tiab] OR Sprint*[tiab] OR “high speed running”[tiab] OR “very-high speed running”[tiab] OR “very high speed running”[tiab] OR Workload[Mesh] OR “Physical Exertion”[Mesh] OR “Heart Rate”[Mesh] OR Acceleration[Mesh] OR Deceleration[Mesh] OR Running[Mesh]) AND (Adolescen*[tiab] OR Young[tiab] OR Youth[tiab] OR Talent*[tiab] OR Junior[tiab] OR Collegiate[tiab] OR adolescent[Mesh] OR “Young Adult”[Mesh]) AND (Soccer[tiab] OR Football[tiab] OR “soccer player*”[tiab] OR “football player*”[tiab] OR Soccer[Mesh])

## Appendix E

Below is the search string for ProQuest Dissertations & Theses A&I.

abstract((Context* OR “Contextual factor*” OR Situation* OR Environment* OR Ecological OR “Social load” OR “Social Stress” OR “Social support” OR “Psychological Stress” OR “Psychological Load” OR “Psychosocial stress” OR “Psychosocial Load” OR “daily life” OR “Daily lives” OR “life stress” OR “life load” OR “Match related” OR “Match-related” OR “Match location” OR “Match outcome” OR Opposition Or Opponent* OR School OR Universit* OR College OR Academic* OR Education* OR Peer OR Peers OR Friend* OR Family OR Parent OR Parents OR Sibling* OR Coach* OR Staff OR Manager* OR Trainer* OR teammate* OR “Social Media” OR “Screen time” OR “dual career” OR “dual-career” OR Work OR profession) AND (“Training load” OR “Internal Load*” OR “External Load*” OR Load OR RPE OR dRPE OR “differential RPE” OR Exertion OR “Heart rate” OR TRIMP OR iTRIMP OR Speed OR Velocit* OR “Speed Zone*” OR Distance* OR Acceleration* OR Deceleration* OR Sprint* OR “high speed running” OR “very-high speed running” OR “very high speed running”) AND (Adolescen* OR Young OR Youth OR Talent* OR Junior OR Collegiate) AND (Soccer OR Football OR “soccer player*” OR “football player*”)) OR title((Context* OR “Contextual factor*” OR Situation* OR Environment* OR Ecological OR “Social load” OR “Social Stress” OR “Social support” OR “Psychological Stress” OR “Psychological Load” OR “Psychosocial stress” OR “Psychosocial Load” OR “daily life” OR “Daily lives” OR “life load” OR “Match related” OR “Match-related” OR “Match location” OR “Match outcome” OR Opposition Or Opponent* OR School OR Universit* OR College OR Academic* OR Education* OR Peer OR Peers OR Friend* OR Family OR Parent OR Parents OR Sibling* OR Coach* OR Staff OR Manager* OR Trainer* OR teammate* OR “Social Media” OR “Screen time” OR “dual career” OR “dual-career” OR Work OR profession) AND (“Training load” OR “Internal Load*” OR “External Load*” OR Load OR RPE OR dRPE OR “differential RPE” OR Exertion OR “Heart rate” OR TRIMP OR iTRIMP OR Speed OR Velocit* OR “Speed Zone*” OR Distance* OR Acceleration* OR Deceleration* OR Sprint* OR “high speed running” OR “very-high speed running” OR “very high speed running”) AND (Adolescen* OR Young OR Youth OR Talent* OR Junior OR Collegiate) AND (Soccer OR Football OR “soccer player*” OR “football player*”))

## Appendix F

Below is the search string for SportRxiv.

Context* OR “Contextual factor*” OR Situation* OR Environment* OR Ecological OR “Social load” OR “Social Stress” OR “Social support” OR “Psychological Stress” OR “Psychological Load” OR “Psychosocial stress” OR “Psychosocial Load” OR “life stress” OR “daily life” OR “Daily lives” OR “life load” OR “Match related” OR “Match-related” OR “Match location” OR “Match outcome” OR Opposition Or Opponent* OR School OR Universit* OR College OR Academic* OR Education* OR Peer OR Peers OR Friend* OR Family OR Parent OR Parents OR Sibling* OR Coach* OR Staff OR Manager* OR Trainer* OR teammate* OR “Social Media” OR “Screen time” OR “dual career” OR “dual-career” OR Work OR profession) AND (“Training load” OR “Internal Load*” OR “External Load*” OR Load OR RPE OR dRPE OR “differential RPE” OR Exertion OR “Heart rate” OR TRIMP OR iTRIMP OR Speed OR Velocit* OR “Speed Zone*” OR Distance* OR Acceleration* OR Deceleration* OR Sprint* OR “high speed running” OR “very-high speed running” OR “very high speed running”) AND (Adolescen* OR Young OR Youth OR Talent* OR Junior OR Collegiate) AND (Soccer OR Football OR “soccer player*” OR “football player*”
